# Under Cover of Darkness, Caterpillars Take Flight: The Immature Stages and Feeding Ecology of the Glasswinged Butterfly, *Oleria baizana* in Eastern Ecuador

**DOI:** 10.1673/031.012.10601

**Published:** 2012-09-05

**Authors:** Thomas R. Walla, Harold F. Greeney

**Affiliations:** ^1^ Colorado Mesa University, Department of Biology, Western Colorado Center for Tropical Research, 1100 North Avenue, Grand Junction, CO 81501; ^2^ Yanayacu Biological Station and Center for Creative Studies, Cosanga, Ecuador, c/o 721 Foch y Amazonas, Quito, Ecuador

**Keywords:** Andes, *Brugmansia*, egg, lthomiinae, lthomiini, larva, Lepidoptera, Oleria, parachuting, pupa, Solanaceae, trenching, vein-cutting

## Abstract

This paper describes the morphology and behavior of the immature stages of *Oleria baizana* (Haensch) (Lepidoptera: Nymphalidae) from northeastern Ecuador. *Brugmansia aurea* Lagerh. (Solanales: Solanaceae) is the larval food plant. Eggs are laid singly, off of the host plant in the leaf litter. During the night, larvae climb a food plant seedling and sever a leaf petiole, parachuting with the leaf to the ground where they remain while feeding. *Oleria baizana* has five larval stadia, and individuals take 77 days to mature from oviposition to adult stage.

## Introduction

The nymphalid butterfly tribe Ithomiini, known for their slow flight, elegant glass wings, and association with Mullerian mimicry rings, has enjoyed a wealth of popular interest and scientific research. All of the adults are likely unpalatable to predators, and many of those investigated rely on dehydropyrrolizidine alkaloids collected by the males from Asteraceae or Boraginaceae plants to obtain their chemical protection (Brown 1984, 1985). The taxonomic diversity and ecological interactions of the Ithomiini have brought them considerable attention as evolutionary models for the process of natural selection and speciation (Bates 1862; Muller 1879; Drummond 1986; Whinnet et al. 2005; Elias et al 2009). Although larvae of some related species feed on Apocynaceae, Ithomiini larvae are best known for their specialized feeding on Solanaceae, a family rich in secondary compounds (Brown and Enriques 1991). Species—specific host associations among the Ithomiini have provided ample material for tests of Ehrlich and Raven's (1965) plant-herbivore coevolution model and have shown that the Ithomiini have likely diversified onto a great number of niches provided by Solanaceae with little evidence of co-cladogenesis (Brown 1985, 1987; Drummond 1986; Wilmot and Freitas 2006). Thus, there is great interest in understanding the details of how Ithomiini species have adapted to the available niches of their solanaceous hosts. Additionally, detailed documentation of larval morphology and behavior has proven useful for testing phylogenetic hypotheses (Brown and Freitas 1994; Wilmot and Freitas 2006).

*Oleria baizana* (Haensch) (Lepidoptera: Nymphalidae) was originally described as *Leucothyris baizana* Haensch, a genus subsequently synonymized with *Oleria* by Haensch (1905). Brown and Freitas (1994) erected the genus *Ollantaya*, placing *O. baizana* in it along with *Ithomia canilla* and *Ithomia aegineta. Ollantaya* was, however, later synonomized with *Oleria* by Lamas (2004). Adults are common at 2000 m in Napo province at Yanayacu Biological Station, but the larval host was previously unknown despite extensive larval collections in the area. Here we add to the growing literature on Ithomiini natural history and present the first description of the immature stages and larval behavior of *O. baizana* from observations at 2000 m in northeastern Ecuador.

## Materials and Methods

All rearing and field investigations were carried out at the Yanayacu Biological Station and Center for Creative Studies (00° 35.949 S, 77° 53.403 W), located in Napo Province in the Andes of northeastern Ecuador. The study site is located approximately five kilometers west of the town of Cosanga, and includes around 2000 hectares of primary cloud forest bordered by cattle pastures and other disturbed habitats (see Valencia 1995). Rainfall averages between 2500–3500 mm per year with a drier season from August to January. Mean monthly temperatures range from 15–17 °C and the general climate is best described as cool and rainy. Larvae were collected at 2000–2100 m and were reared in individual plastic containers at the onsite ambient research lab, located at 2150 m. Fresh leaf material was added to containers every two days. Larval measurements were taken at the beginning and end of each stadium.

As part of a broader study focused at Yanayacu Biological Station (Caterpillars and Parasitoids of the Eastern Andes), more than 25,000 caterpillars have been collected from plants using both exhaustive collection methods in plots and intensive caterpillar searching along trails and roads. This database provided a reference for determining the host plant breadth of *O. baizana*.

An oviposition event was observed and photographed by TRW, and the egg was reared to third instar. Plants and leaf litter were then intensively searched for larvae during a two-day period. Six larvae were observed closely for life history information and 15 larvae in various instars were placed on three small (0.5 m) host plant individuals that were transplanted from the field to pots on the porch of the biological station for behavioral observations. Such observations were made on a casual basis at different hours of the night and day.

## Results

**Figure 1.**
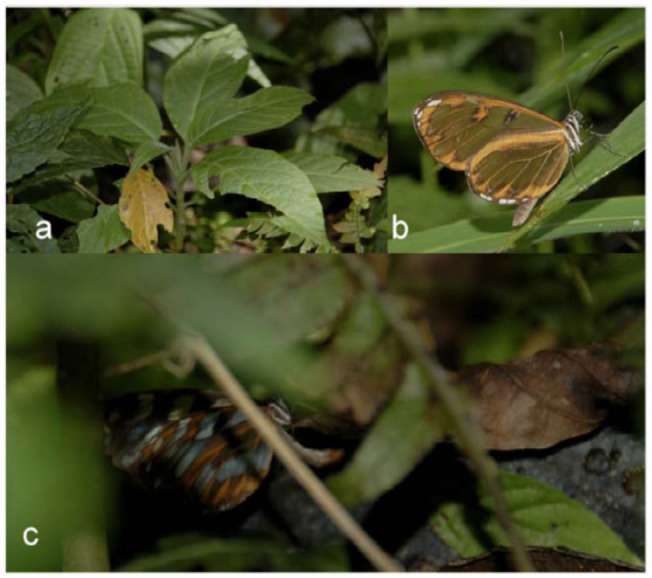
(a) *Brugmansia aurea* host plant of typical size for *Oleria baizana* caterpillars; (b) Adult male *Oleria baizana;* (c) *Oleria baizana* female ovipositing on a dead leaf on the forest floor near the host plant. High quality figures are available online.

**Oviposition behavior.** At 13:30 a female was observed flying slowly through the forest understory, remaining 1–2 m above the ground in the vicinity of a stream. She periodically landed on a variety of understory plants, rapidly drumming her forelegs on leaves. When she encountered a young (∼0.5 m tall) individual of *Brugmansia aurea* Lagerh. (Solanales: Solanaceae) (Figure 1a), she landed briefly on several leaves and then dropped to the leaf litter under the plant. After walking on the plant stem and several dead leaves, she oviposited on the underside of a curled dead leaf (of a species different from the host) approximately 10 cm from the stem (Figure 1c) and then flew briskly away from the area. A search of dead leaves under this plant found one second and one third instar *O. baizana*.

**Larval feeding behavior: caterpillars take flight.** The search for larvae on multiple outings yielded more than 20 individuals representing all five instars. Larvae of all instars were most frequently found feeding or resting on the underside of recently fallen leaves on the forest floor under small individuals (< 1 m) of the host. They were also found on the underside of dead leaves under the canopy of the same plants, resting in the “J” position typical of many ithomiine larvae (Wilmot and Freitas 2006). Despite intensive searching on host plants as part of a broader caterpillar inventory at the study site and specifically as part of this investigation, larvae were never encountered feeding on host leaves still attached to the plant. Inspection of host plants showed leaves on the forest floor appeared to have been severed from the host plant midway along the petiole and had not senesced naturally from the plant. This observation led to a closer inspection of caterpillars that had been placed on potted plants.

Periodic observations showed that during daylight hours caterpillars remained on the underside of fallen leaves under plants and fed inconspicuously. In the darkness of the early morning (04:00), a fourth instar climbed the stem of the plant, wandered across a few leaves, then steadily chewed through one of the petioles, gliding with the leaf to the litter under the host.this leaf—cutting behavior was subsequently observed by large fourth instars as well as by much smaller early third instars. In all cases, larvae ascended the plant to cut new leaves when the leaf they were feeding on was completely or nearly consumed. Prior to cutting, larvae wandered onto several leaves before returning to the petiole. Then, with their head facing the plants stem, they began chewing the petiole in a straight cut across the vascular tissue. The entire process of cutting through the petiole took less than 20 minutes for a fourth instar and slightly more than 35 minutes for a small third instar. In both of the complete cuttings observed, when the final cut was made, leaf and caterpillar together glided to the litter. In one case the caterpillar continued to chew on the severed petiole, perhaps feeding on the soft tissue and clear exudates. In other instances, caterpillars in the leaf litter were found chewing on the recently cut petiole or cutting the petiole again within a couple centimeters of the first cut.

**Figure 2.**
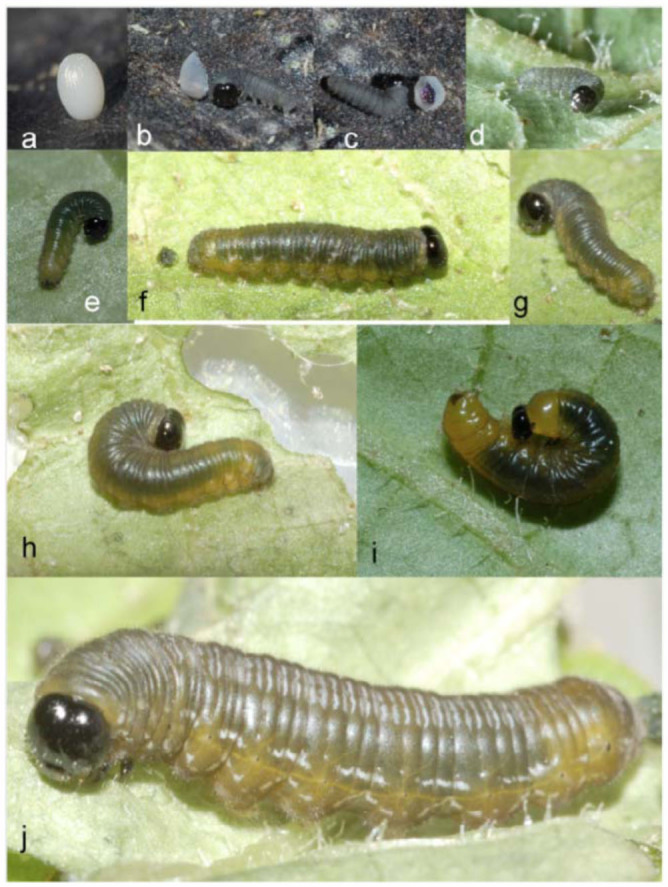
Early stages of *Oleria baizana* in eastern Ecuador, (a) egg; (b-c) first instar moments after hatching with partially consumed egg; (d-g) first instar; (h) second instar; (i) second instar pre-molt; (j) third instar. High quality figures are available online.

**Egg** (Figures 2a-c; n = 1; diameter approx. 1 mm, height 1.5 mm; development time = 12 days). White, barrel-shaped, smoothly tapered to oval at the top, turned brown before hatching; surface covered with vertical rows of divot-like sculpturing; laid singly (n = 1); upon emergence, larvae consumed two thirds of the egg shell.

**First instar** (Figures 2b-g; n= 1; body length = 2.5–5.0 mm; development time = 7 days). Head capsule sub-quadrate, slightly broader at base, with a shallow epicranial notch; head surface shiny, dark brown, bearing a few sparse, long, pale setae; body parallel-sided, each segment slightly produced laterally; body light gray, with green gut contents showing through later in instar, body sparsely covered with long soft setae the length of 1/3 head capsule; J-position at rest.

**Second instar** (Figures 2h-i; n = 2; body length = 9 mm; development time = 5 days). Head capsule similar to first instar, with a weak epicranial notch; head dark shiny brown with sparse setae; body surface covered in pilosity of very short, soft setae, proportionately much shorter than those in the first instar; color two-toned, with dorsum dark olive green separated by a weak yellow spiracular line from dark yellow venter and legs.

**Figure 3.**
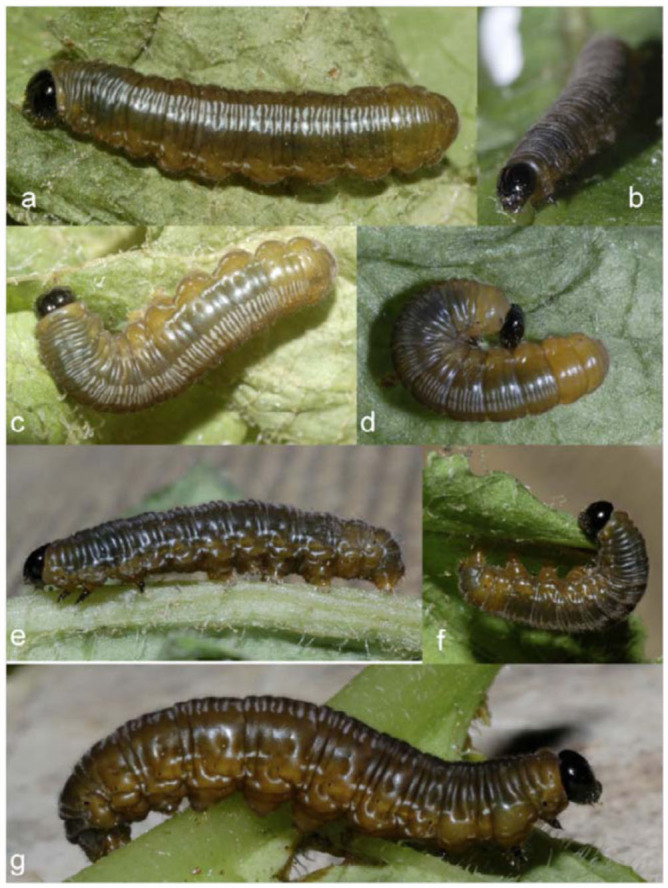
Early stages of *Oleria baizana* in eastern Ecuador, (a-c) third instar; (d) third instar pre-molt; (e-g) fourth instar. High quality figures are available online.

**Third instar** (Figures 2j, 3a-d; n = 5; body length = 9.5 mm; development time = 5-8 days). Head and body shape similar to late second instar, head dark brown sparsely covered with short setae approx 1/10 width of head capsule, T1 dark yellow in color, T2-A9 dorsum dark olive green divided from dark yellow ventrolaterally by a fine faint yellow spiracular line extending from T1 to A10, faint blue-gray pairs of parentheses shaped transverse lines on dorsum of segments A1– A9 become apparent in this instar giving a glossy appearance to the dorsum, evenly distributed pubescence covering the body with longer subventral setae protruding from proleg bases, and A10 margin. Spiracles dark in color, T1 and A10 spiracles larger in size than others. A pair of discontinuous blue-gray wavy lines from A1 to A10 bracket the spiracles causing a glossy appearance in lateral view.

**Fourth instar** (Figures 3e-g; n = 12; body length = to 35 mm; development time =11 days). Head and body similar to late third instar; head capsule dark brown with pronounced epicranial notch, sparse short setae covering head; T1 dark yellow, T2-A8 dorsum dark olive green divided from dark yellow ventrolaterally by fine faint yellow spiracular line from T1 to A10, evenly distributed pubescence covering body with longer subventral setae protruding from proleg bases and A10 margin. Spiracles dark brown forming small points with a T1 and A10 forming larger dark spot, Transverse blue-gray parentheses shaped lines on dorsum of T3-A9 more apparent compared to third instar. Light blue-gray discontinuous markings dorsal and ventral to spiracles forming faint dorsolateral striping pattern more apparent compared to third instar.

**Figure 4.**
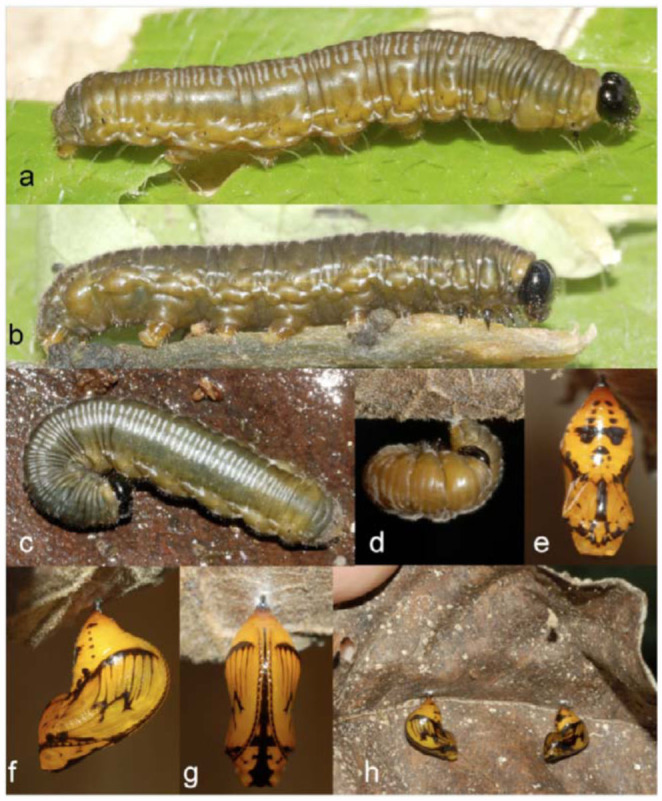
Early stages of *Oleria baizana* in eastern Ecuador, (a-b) fifth instar; (c) fifth instar in “J position” under a dead leaf; (d) prepupa; (e) pupa dorsal view; (f) pupa ventral view; (g) pupa lateral view; (h) two pupae under a dead leaf in the leaf litter. High quality figures are available online.

**Fifth instar** (Figures 4a-c; n = 1; body length = to 21 mm; development time =13 days including prepupa). Head and body similar to fourth instar, head capsule dark brown with pronounced epicranial notch, sparse short setae covering head; T1 dark yellow, T2-A8 dorsum dark olive green divided from dark yellow ventrolaterally by fine faint yellow spiracular line from T1 to A10, evenly distributed pubescence covering body with longer ventrolateral setae protruding from proleg bases and A10 margin; spiracles dark brown forming small points with a T1 and A10 forming larger dark spots; transverse blue-gray parentheses shaped lines on dorsum T3-A10 more apparent compared to fourth instar; light blue-gray discontinuous markings dorsal and ventral to spiracles forming faint dorsolateral striping pattern more apparent compared to fourth instar.

**Pre—pupa** (Figure 4d; n = 1; development time = 4 days). Pre-pupal larva becoming brownish yellow and somewhat translucent; larva spins a white silk pad.

**Pupa** (Figures 4e-h; n = 1; development time = 18 days). Larvae pupated under curled dead leaves on the forest floor; pupa pendant, angle between abdomen and thorax 120° (“pupal angle” of Willmott and Freitas 2006), body translucent dark yellow with black markings, cremaster stalk black, silk patch white, wing cases with black shading in some cells forming thin vein-like stripes, fused submarginal spots forming black line at wing margin, paired dorsolateral bands on abdomen formed by rows of dark black spots, proboscis black, casing over antennae segments black forming long black dashed lines.

## Discussion

### Systematics

An extensive phylogenetic analysis of the Ithomiini and related species using both immature and molecular characters (Wilmot and Freitas 2006) supported the genus *Oleria* as monophyletic. The authors further commented that, based on unpublished data from Wilmott and A. Whinnett, both molecular and morphological evidence suggests *O. baizana* and *O. santineza* are closely related within *Oleria*. A recent comprehensive molecular phylogenetic analysis of the Oleriina subtribe by De-Silva et al. (2010) supported this by the placement of *O. baizana* in the *Makrena* species group, one of four well-supported species groups comprising the *Oleria* genus. That study supported *O. baizana* as the sister species to *O. radiana* forming a sister clade to *O. santineza* + *O. fumat*. Our observations of larval morphology, cryptic, dead-leaf pupal coloration, and off-plant oviposition behavior also lend support to a close relationship of *O. baizana* and *O. santineza*. The drab olive coloration of the larvae and the dark yellow cryptic coloration of the *O. baizana* pupae may be derived characteristics among the Oleriina shared with *O. santineza* and others of this lineage. Wilmot and Freitas (2006) found green pupal background color to be an unambiguous synapomorphy for Oleriiini, though they note some species in their analysis were missing data and others showed a different derived state such as *O. santineza*, which is orange in color, similar to *O. baizana*. Though Wilmot and Freitas (2006) included larval characters in their analysis of the Ithomiini, they did not specifically score larval color pattern as it pertains to crypsis; thus, inferences regarding the timing of leaf litter crypsis evolution is limited. However, available color images from Ithomiini larvae presented by Wilmot and Freitas (2006) indicate the group shows a great diversity of color patterns ranging from cryptic green to black and yellow banding, most of which are either aposematic or cryptic on green leaf surfaces.

Wilmott and Freitas (2006) list 10 unambiguous synapomorphies for *Oleria*, only two of which are immature characteristics. For the *egg relative size* character, four *Oleria spp*. eggs scored > 2.4 (0), and for the pupal characteristic 61 *lateral tubercles at junction between wing base and posterior edge mesothorax*, they scored absent (0). Our immature *O. baizana* showed the same character states as other members of the group supporting its position in *Oleria*. Wilmott and Freitas (2006) also listed four ambiguous synapomorphies for which *O. baizana* showed some variance from the cited state, but shared all states with *O. santineza* that were included in that study. Character 54 *pupal angle* was 120 degrees. For pupal character 65 *reflective areas O. baizana* scored 0 with no reflective areas. For pupal character 66 *color of the cremaster stalk* our specimens scored black (0), which differed from the red to pinkish state cited for the genus. For pupal character 54 *paired dorso*— *lateral patterned bands on abdomen*, we found them to be the same color as the background pupae color state 0. Comparisons to images in Wilmott and Freitas (2006) showed that both *O. baizana* and *O. santineza* have pupae with a black cremaster stalk, which is contrary to the predominant red to pinkish pattern reported for most *Oleria*.

### Behavior

The remarkable behavior exhibited by *O. baizana*, whereby it chews through the petiole and parachutes to the forest floor to feed in the obscurity of the leaf litter, is to the best of our knowledge unique within the Lepidoptera. Because the short descent to the forest floor does not involve the conversion of gravitational energy to aerodynamic work, we classify it as parachuting (*sensu* Dudley et al. 2007). A similar parachuting behavior was reported for the nettle caterpillar *Scopelodes contracta* (Yamazaki 2010), in which larvae snip the petiole and fall with their leaf to the ground where they burrow into the soil and pupate. This behavior is distinct from that observed in *O. baizana* in that it does not involve feeding on the host on the ground, nor does it require the return of the caterpillar to the host plant to harvest another leaf. Instead evidence suggests the leaf is snipped to slow the descent that may be several meters, but is subsequently abandoned. In contrast, the primary advantage of snipping for *O. baizana* appears to be a change of feeding venue from arboreal to leaf litter, and perhaps a reduction in the toxicity of leaf compounds resulting from the cut leaf veins.

Many species of insects are known to trench and cut veins or petioles to the point of wilting, but all reports indicate these species remain on the plant to feed (reviewed in Dussourd and Denno 1993). The most common explanation for caterpillars cutting leaf veins and tissues proximal to the feeding area is that this action reduces the flow of physical (latex) and chemical plant exudates to the area where feeding will occur, a behavior that is particularly common among species feeding on latex rich host plants (Dussourd and Denno 1991, 1994; Dussourd 2009). These authors experimentally demonstrated that the benefits of trenching are a reduction in latex pressure, which may act as a physical deterrent to herbivores and a reduction in quantity of toxins delivered to the area of damaged tissue. Petiole cutting to the point of wilting has been considered an extension of vein cutting and trenching behavior carried out closer to the base of the leaf (Dussourd 1999). Although *Brugmansia* does not produce latex, it is possible that complete petiole cutting in *O. baizana* reduces the flow of secondary compounds to the area where larvae feed. While few Ithomiini species have been observed to trench, there are some notable exceptions. Wilmot and Freitas (2006) inferred Apocynaceae as a likely ancestral host for the Ithomiini. Other members of the Danainae *(sensu* Wahlberg et al. 2005) feed on Apocynaceae, and many species in this group trench or vein cut to restrict the flow of latex (Ackery 1987; DeVries 1987; DeVries 1991; Ackery and Vane-Wright 1984). The members of the closely related Tithoreini tribe feed on Apocynaceae, with vein-cutting observed in *Aeria eurimedea* (DeVries 1987). Vein cutting or trenching has also been observed in members of the more derived genera *Mechanitis* (Young and Moffett 1979) and *Melinea* (DeVries 1987), which feed on Solanaceae (reviewed in Dussourd 1993). It appears likely that vein cutting behavior is an ancestral trait in the Ithomiini that was lost with the colonization of Solanaceae but has resurfaced independently in several species including *O. baizana*.

By completely removing their food from the host plant, *O. baizana* larvae modify two major components of their life history: they dissociate the leaf from its source for toxins and deterrents, and they escape the potential exposure to predators and parasitoids specialized in arboreal hunting. Accordingly, the coloration of *O. baizana* larvae and pupae are well camouflaged in the leaf litter, showing none of the bright green, yellow, and sometimes metallic patterns common among the early stages of other *Oleria* species that feed in the mid-forest levels. The tribes Tithoreini, Methonini, Melinaeini, and Athesis+Patricia (*sensu* Wilmot and Freitas 2006) are known to have brightly colored larvae and members of the Tithoreini have been shown to sequester toxins from their hosts (Trigo and Brown 1990), rendering them unpalatable to some predators. The remaining tribes of Ithomiinae have larvae and pupae that are generally cryptic among the green leaves of living plants. The dead leaf crypsis evident in the larvae and pupae of *O. baizana*, as well as its close relative *O. santineza* (Wilmot and Freitas 2006), suggest a shared shift in feeding niche away from the leaves of living plants and toward leaf litter microhabitats.

We speculate that specialized leaf-cutting behavior and adaptations to the leaf litter microhabitat have evolved in response to the top-down forces of predation/parasitism on arboreal caterpillars, fueling a shift to the relatively enemy-free space of the leaf litter. Most of the literature on petiole snipping and trenching behaviors suggests they evolved to protect caterpillars from plant toxins (Dussourd 1993). While this may be the case for *O. baizana*, the rarity of snipping or trenching among derived Ithomiini suggests additional factors may be at play. By completely severing the leaf instead of allowing it to hang by the last tissues as in some species, larvae risk being unable to relocate their host. It follows that some advantage is gained by dropping from the plant after the flow of plant toxins has been stopped, and that this outweighs the risk of losing the host. In addition, the off-plant oviposition behavior provides further evidence of an advantage to not being associated with the host plant itself despite the risk of first instars failing to encounter their host. These two lines of evidence are further supported by the nocturnal timing of petiole snipping, which also suggests diurnal predator avoidance. While protection from leaf chemistry cannot be ruled out as a factor driving petiole snipping, it appears likely that in this case it provides an escape from top down forces on the plant.

As a curious side-effect, leaf cutting may constrain larvae to feeding on very young host plants. Larvae were found feeding only on short plants (< 1 m), where petiole cutting would be unlikely to drop individuals more than a few centimeters from their host. Presumably larvae separating leaves from taller plants would risk drifting too far from the trunk and their only source of fresh leaf material. Indeed, exhaustive searching on mature plants (2–3 m tall) yielded no caterpillars and no evidence of severed petioles. As a seedling specialist, *O. baizana* may influence host plant distribution and thereby tree community diversity according to the Janzen-Connell seed dispersal hypothesis (Janzen 1970; Connell 1971). Densitydependent herbivory by a specialist herbivore may negatively affect recruitment of seedlings near the parent plant where seed density is highest, preventing clumped distributions of trees and promoting species coexistence.

To the best of our knowledge, the parachuting behavior followed by leaf litter feeding of *O. baizana* is the first to be documented among Lepidoptera. The rarity of this behavior in the literature may be due in part to a natural bias of lepidopterists searching for caterpillars on living plant tissue. It stands to reason that the habit of feeding on recently fallen leaves among the dead and decomposing leaves of the forest floor excludes this species from the sampling methods of most large scale caterpillar rearing projects (e.g., Janzen and Hallwachs 2009), including the project that led to the rearing of this caterpillar (Dyer et al 2011). We suggest there is great value in the careful documentation of individual life histories that complement the large scale rearing efforts in place around the globe.

**Figure 5.**
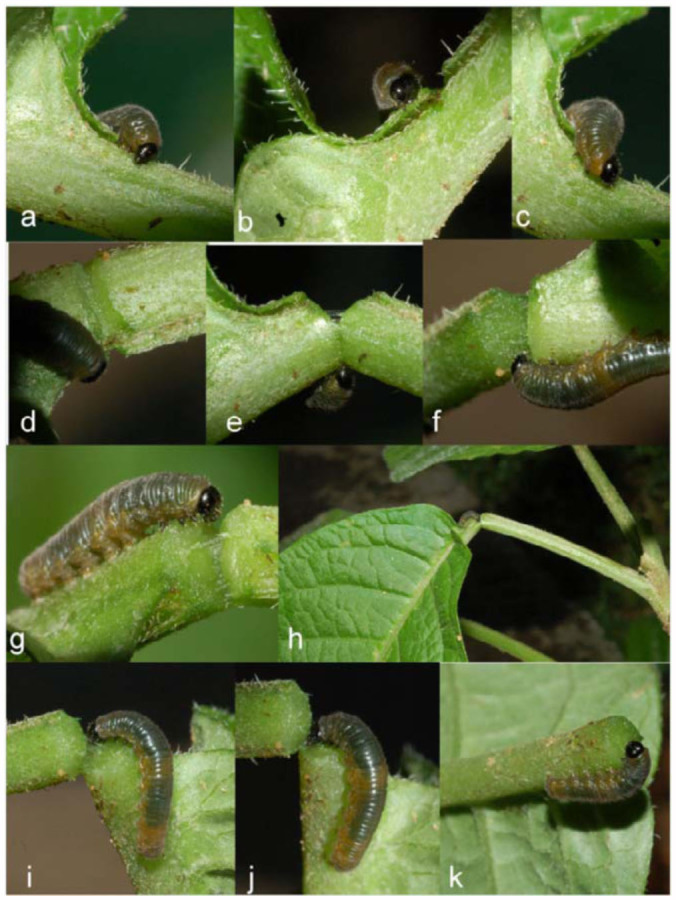
Third instar *Oleria baizana* in eastern Ecuador cutting a leaf petiole, (a-j) sequential record of third instar cutting leaf petiole; (k) third instar feeding on exudates of cut petiole after falling to the ground. High quality figures are available online.

**Figure 6.**
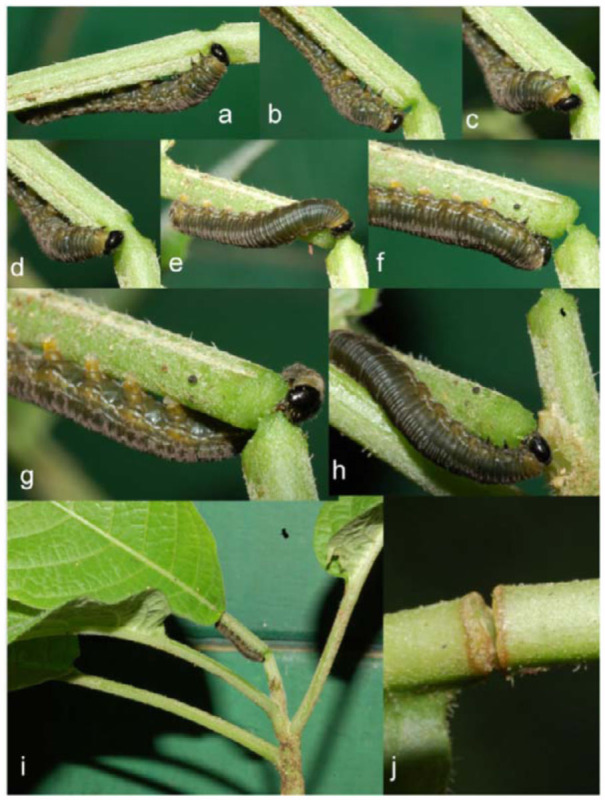
Fourth instar *Oleria baizana* in eastern Ecuador cutting a leaf petiole, (a-h) sequential record of fourth instar cutting leaf petiole; (i) fourth instar cutting a leaf petiole; (j) scar on partially severed leaf petiole. High quality figures are available online.
